# Assessment of Growth, Lipid Metabolism and Gene Expression Responses in Senegalese Sole Larvae Fed With Low Dietary Phospholipid Levels

**DOI:** 10.3389/fphys.2020.572545

**Published:** 2020-09-30

**Authors:** Ismael Hachero-Cruzado, Ana Rodriguez-Rua, Ivana Torrent, Javier Roman-Padilla, Manuel Manchado

**Affiliations:** ^1^IFAPA Centro El Toruño, Junta de Andalucía, El Puerto de Santa María, Spain; ^2^“Crecimiento Azul”, Centro IFAPA El Toruño, Unidad Asociada al CSIC, El Puerto de Santa María, Spain; ^3^Centro Oceanográfico de Cádiz, Instituto Español de Oceanografía, Cádiz, Spain

**Keywords:** phospholipids, Senegalese sole, fish larvae, microdiets, apolipoproteins

## Abstract

Phospholipids (PL) are essential molecules for larval growth and development. In this study, growth, lipid metabolism and gene expression responses associated with different dietary PL levels in pelagic sole larvae were evaluated. In a first trial, the long-term effects on growth and survival of two experimental microdiets (MD) containing high (High-PL) or low (Low-PL) PL levels were tested and compared to a diet based on live prey (rotifers). The MD were supplied from 3 to 10 days post-hatch (dph) and *Artemia* from day 8 to 29 dph. High-PL fed larvae had higher dry mass (1.2-fold) than Low-PL fed larvae at 8 dph and both MD were smaller (2.9-fold) than larvae fed live preys. However, a compensatory growth (33% between 8 and 20 dph) occurred when MD were substituted by *Artemia* and by the end of the trial no significant differences in mass or survival occurred between the dietary treatments. In a second trial, growth, lipid metabolism and gene expression profiles of larvae fed with MD up to 8 dph were analyzed. Growth data confirmed that mass of larvae fed with High-PL was higher (1.3-fold) than the those fed Low-PL and they had lower levels of triacylglycerol (2.8-fold), cholesterol (1.2-fold) and cetoleic acid (1.7-fold). Histological analysis indicated an excess of lipid vacuoles in larvae fed with Low-PL and the expression analysis revealed a coordinated response to enhance lipid mobilization since the expression of genes involved in PL intermediate synthesis, PL remodeling as well as eight apolipoprotein was up-regulated. The down-regulation of apolipoprotein *apob2* in larvae fed with Low-PL indicated a specific regulation by PL levels. The present work provides insight into the responses associated with dietary PL in early fish larvae, which will be of use for future studies aimed as designing effective larval sole diets.

## Introduction

Lipids are essential molecules in larval physiology supplying not only the energy required for growth but also participating in membrane formation and synthesis of signaling molecules. Triacylglycerol (TAG) is the main lipid class responsible for energy provision and fatty acid (FA) delivery to the cells (Mourente and Vazquez, 1996; Hamre et al., 2013; Hachero-Cruzado et al., 2014). As an energy supply molecule, dietary TAG levels are key effectors of larval development and tightly modulate FA and TAG biosynthesis pathways to provide sufficient energy for an optimal growth (Hachero-Cruzado et al., 2014). Other important lipid classes are the phospholipids (PL) with a major structural role in the assembly of cell membranes and tissue formation (Niu et al., 2008; Tocher et al., 2008; Saleh et al., 2013). Moreover, PL are an integral part of lipoproteins and play a key role in lipid transport (Chapman, 1980; Babin and Vernier, 1989) and occasionally act as a source of energy during embryogenesis and early larval development (Rainuzzo et al., 1992; Rønnestad et al., 1995; Mourente and Vazquez, 1996). Microdiets (MD) represent an experimental methodology suited for evaluation the effect of dietary compounds on fish larvae and avoid ambiguous results caused by the metabolic transformations that can occur if live preys are used as the vehicle (Tocher et al., 2008; Cahu et al., 2009; Hamre et al., 2013). This is especially relevant when the aim is to evaluate structural molecules such as PL and live preys tend to maintain their levels constant making difficult to establish a good gradient between the dietary treatments (Hachero-Cruzado et al., 2014; Roman-Padilla et al., 2017).

It is well-accepted that marine larvae require a supply of lipids from the diet for growth and development as lipid metabolism is still not fully-functional. Hence, a good understanding of the mechanisms involved in lipid transport and fat accumulation in the intestine under different lipid levels is required since these two factors are a constraint for nutrient trafficking in early fish stages (Fontagné et al., 1998; Morais et al., 2007; Gisbert et al., 2008). Several authors have reported that an optimal ratio between PL and neutral lipids is required to prevent the negative effects of lipid accumulation in the intestine and this requires more knowledge about the larval biosynthetic capacity and the endogenous capacity for lipoprotein formation and lipid transport that are highly dependent on intestine maturation and age (Deplano et al., 1991; Diaz et al., 1997; Gisbert et al., 2008; Carmona-Antoñanzas et al., 2015). In a previous study using Senegalese sole larvae we demonstrated that early larval stages modified cell regulatory pathways as a function of dietary TAG levels to mitigate excess lipid accumulation in the intestine (Hachero-Cruzado et al., 2014; Roman-Padilla et al., 2017). These experiments were carried out using dietary PL levels higher than those recommended for fish larvae (Tocher et al., 2008). Thus, new research is required in sole to understand the physiology of intestinal lipid transport under suboptimal dietary PL and to identify compensatory responses triggered by the larvae when the PL/TAG ratio is modified.

Phospholipid biosynthesis in fish larvae is highly dependent on intestine maturity and liver function. Diaz et al. (2002) reported that early fish larvae use biliary lipids to synthesize the first set of lipoproteins. In mammals, two phosphatidylcholine (PC) synthetic pathways are present in the intestine, (i) the remodeling pathway by acylation of dietary lysoPC (LPCAT) and (ii) *de novo* synthesis by CDP-choline-dependent (CPT) pathway. Moreover, the phosphatidylethanolamine N-methyltransferase (PEMT) pathway appears to be liver-specific (Mansbach and Gorelick, 2007). In fish, all the enzymes for the endogenous production of PC are present in the liver and intestine. However, the activity of the PEMT pathway *in vivo* using animals has not been tested (Oxley et al., 2007; Carmona-Antoñanzas et al., 2015). Interestingly, expression levels of the main enzymes involved in PL biosynthesis were lower in the intestine of salmon fry compared to par, supporting the idea that the developmental status of the intestine determines lipid transport. In this study, the effects on growth and the physiological and expression responses that occur in early larval stages of the flatfish Senegalese sole fed a MD with a low PL/TAG ratio with suboptimal total PL content compared with larvae fed with a control MD (high PL levels) were investigated. In a first trial, long-term effects of larvae fed with both MD on growth and survival were determined and results were validated using a diet based on live prey (rotifer). Since clear differences in growth associated with MD were found a second trial was performed to validate growth results and identify the specific physiological and gene expression responses associated with each MD. Changes in lipid classes, FA profile, histological characterization of the intestine and liver and gene expression patterns were determined. This multidisciplinary approach provided new clues about the role of PL levels in early larval stages of marine fish and the compensatory mechanisms triggered in the larvae in response to dietary PL levels.

## Materials and Methods

### Experimental Diets and Larval Rearing

The inert diets were supplied by Sparos Lda. (Olhão, Portugal). Ingredients used in the preparation of MD are indicated in Table 1. Both MD (named as Low-PL and High-PL) were isonitrogenous and isolipidics with a similar FA profile (Table 2). The main differences in the MD were the PL/TAG ratio (Low-PL 0.05 or High-PL 0.73% dry mass (DM), respectively) and PL content (0.53 and 4.97%, respectively) (Table 2). The marine-derived ingredient Phosphonorse (Tromsø Fiskeindustri, Norway) rich in PL (40% DM) was used as the main PL source in the diet. Secondary sources for PL were plant derived ingredient (Micalamix (Sparos Lda); PL: 1.2% DM), squid meal (PL: 1.2% DM), fish meal (PL: 1.1% DM) and CPSP 90 (marine derived ingredient; PL: 0.8% DM). Pellet diameter for both MD ranged between 100 and 200 μm.

**TABLE 1:** Ingredients composition (% DM) of experimental diets.

Ingredient	Low-PL	High-PL
Squid meal^a^	9.2	9.2
Fish meal^b^	10.0	10.0
CPSP90^c^	9.0	9.0
Micalamix^d^	36.4	36.4
Attractant mix^e^	4.0	4.0
Taurine^f^	1.5	1.5
Wheat meal^g^	0.0	2.0
Binder^h^	6.0	6.0
Fish oil^i^	10.3	0.0
Tuna oil^j^	9.0	0.0
Vevodar®^k^	0.0	0.3
Phosphonorse^l^	0.0	17.0
Premix^m^	2.0	2.0
Vitamin C^n^	0.5	0.5
Vitamin E^o^	0.1	0.1
NaH_2_PO_4_	2.0	2.0
TOTAL	100	100

*Ingredient (g 100 g diet^–1^). ^a^Super Prime without viscera: 82% crude protein (CP), 6% crude fat (CF), Sopropêche, France. ^b^Fishmeal LT70: 71% CP, 13% CF, EXALMAR, Peru. ^c^CPSP 90, 84% CP, 10% CF, Sopropêche, France. ^d^Proprietary SPAROS product for marine fish: 84% CP, 8% CF. ^e^Proprietary SPAROS premixes (amino acid cocktail). ^f^L-Taurine 98.5%: Ajinomoto Eurolysine SAS, France. ^g^Wheat meal: 11.7% crude protein, 1.6% crude fat, Casa Lanchinha, Portugal. ^h^Fish edible gelatine, Lapi Gelatine, France. ^i^Marine oil omega 3: Henry Lamotte Oils GmbH, Germany. ^j^Omegavie Tuna Oil 25 DHA TG, Polaris, France. ^k^ARA-rich oil, DSM Food Specialties, the Netherlands. ^l^Marine phospholipids and marine oils, Tromsø Fiskeindustri A/S, Norway. ^m^PVO40.01 Premix for marine fish, PREMIX Lda, Portugal. ^n^Ascorbil monophosphate, PREMIX Lda, Portugal. ^o^α-Tocopherol, PREMIX Lda, Portugal.*

**TABLE 2:** Proximal composition, lipid classes (in % DM), and FA composition (in % Total FA) of MD with a high PL (High-PL) or low PL content (Low-PL).

Proximal composition	Low-PL	High-PL
Protein	61.40	61.60
Soluble protein	21.20	21.20
Lipids	21.33	21.44
Ash	4.20	4.20
**Lipid classes**		
PC	0.23	3.60***
PE	0.31	0.64
PI	0.07	0.34
PS	0.11	0.39
TPL	0.73	4.97**
CHO	0.23	0.65
TAG	13.38	9.31*
PL/TAG	0.05	0.53***
**Fatty acids**		
MA (14:0)	4.34	4.25
PA (16:0)	16.17	18.30**
SA (18:0)	3.16	2.48*
Total saturated	25.17	26.05
16:1n-9	5.15	4.15***
OA (18:1n-9)	12.22	12.90
VA (18:1n-7)	2.06	3.14
GA (20:1n-9)	6.05	6.53
CA (22:1n-11)	7.22	6.19***
Total monounsaturated	33.82	34.51
LA (18:2n-6)	5.61	7.39
EA (20:2n-6)	0.29	0.18
ARA (20:4n-6)	1.05	1.27*
Total (n-6) PUFA	7.48	9.07
ALA (18:3n-3)	1.34	1.36
ETA (20:4 n-3)	0.48	0.40
EPA (20:5 n-3)	7.21	6.08***
DPA (22:5 n-3)	1.01	0.64
DHA (22:6 n-3)	14.27	12.76
Total (n-3) PUFA	26.59	22.75
Total PUFA	35.04	32.78

Values are expressed as mean (n = 3). Results of Student’s t-test analysis are shown. Asterisks represent significant differences (*P < 0.05; **P < 0.01; ***P < 0.001). PC, phosphocholine; PE, phosphatidylethanolamine; PI, phosphatidylinositol; PS, phosphatidylserine; TPL, total polar lipids; CHO, cholesterol; TAG, triacylglycerol; MA, Myristic acid; PA, palmitic acid; SA, stearic acid; OA, oleic acid; VA, vaccenic acid; GA, gadoleic acid; CA, cetoleic acid; LA, linoleic acid; EA, eicosadienoic acid; ARA, arachidonic acid; ALA, α-linolenic acid; ETA, eicosatetraenoic acid; EPA, eicosapentaenoic acid; DPA, docosapentaenoic acid; DHA, docosahexaenoic acid.

Senegalese sole eggs were provided by the Aquaculture company CUPIMAR (San Fernando, Caìdiz, Spain). They were transported to IFAPA center El Torunþo (El Puerto de Santa Maria, Spain) and incubated at a density of 2,000 egg L^–1^ in a cylindric-conical tank (15 L) with 50% water renewal per hour until larvae were moved to the experimental tanks.

#### Trial to Evaluate the Long-Term Effects of MD on Growth and Survival

In the first trial, three experimental groups were established: one fed with High-PL MD, one fed with Low-PL MD, and one third group that was supplied live prey (LP; rotifer). This last group was used as a reference to monitor that growth and survival rates in groups fed with MD were acceptable.

Sole larvae at 2 days post-hatch (dph) were transferred to nine 80 L tanks (three replicates per treatment) at an initial density of 50 larvae L^–1^. The triplicate tanks per treatment were connected to a closed recirculation system (total water volume 600 L) consisting of a mechanical filter, a skimmer (SC 2060-SC4580, Deltec), ultraviolet lights (Vecton) and a biofilter (Eheim professional 600, typ 2075). Lights were kept off until the onset of external feeding at 3 dph. Thereafter, a 14 Ligth:10 Dark photoperiod with a light intensity of 400 lux was established. Temperature, oxygen and salinity were 20.0 ± 0.3°C, 6 ± 1 ppm and 39 ± 0.4, respectively. Water exchange (∼40% daily) was initiated at 6 dph until 8 dph and thereafter it increased progressively up until 150% by the end of the trial. Tanks were provided with a central draining pipe with a 250 μm mesh and gentle aeration.

**FIGURE 1:**
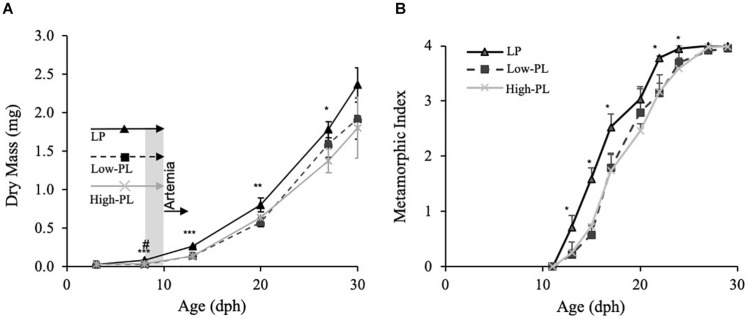
Growth (DM) **(A)** and metamorphic stages **(B)** of Senegalese sole larvae fed live prey (rotifers) enriched with microalgae (LP;black line), MD with a high PL (High-PL, gray line) or a low PL content (Low-PL, dark dashed line). Data are presented as mean ± SD (*n* = 3). Asterisks denote significant differences between MD and LP and # between High-PL and Low-PL MD (Student’s *t* test; *^#^*P* < 0.05; **^##^*P* < 0.01; ***^###^*P* < 0.001). The feeding regimes are indicated in panel A. LP (rotifers), Low-PL and High-PL were supplied from 3 to 10 dph. From 8 to 10 dph (square in gray), there was a co-feeding with *Artemia* metanauplii and from 10 dph to the end of the trial, only *Artemia* was supplied to the three groups. Classification of S0–S4 stages was carried out according to Fernández-Díaz et al. (2001).

Larvae were fed with the MD between 3 and 10 dph. MD were supplied by hand (0.5–1 g per tank day^–1^) five times per day (8:00, 11:00, 14:00, 16:00, and 19:00 h). The LP group was fed with rotifers (*Brachionus plicatilis*) enriched with *Tisochrysis lutea* between 3 and 10 dph. The rotifer (rot) density in tanks ranged between 10 and 20 rot mL^–1^. Microalgae were added daily to the water at a density of 300,000 cells mL^–1^ for *Nannochloropsis gaditana* B3 strain and 50,000 cells mL^–1^ for *Tisochrysis lutea*. At 8 dph, *Artemia* metanauplii were supplied to all larval groups (2 days co-feeding) starting at 0.5 metanauplii mL^–1^ at 8 dph to 5 metanauplii mL^–1^ at 29 dph (Figure 1). Larvae (*n* = 20) were sampled at 3, 8, 11, 13, 15, 17, 20, 22, 24, 27, and 29 dph (8:00) and larval somatic growth and metamorphic stage were determined. Larval dry mass (DM) and the classification of metamorphic stage was carried out as previously described (Fernández-Díaz et al., 2001). The specific growth rate (SGR) was calculated using the following formula SGR (% d^–1^) = (lnW_f_ − lnW_i_)/Δt × 100; where W_f_, W_i_, and Δt represented final and initial mass and time interval, respectively. Final survival rates were calculated as the percentage of fish that survived to the end of the trial compared to the total number at the beginning minus the individuals removed in the sampling.

#### Trial to Evaluate the Effects of MD on Physiology and Gene Expression

The second experimental trial used the same experimental set-up as the first trial but this time it was focused on the pelagic stages from mouth opening (3 dph) to 8 dph when larvae were fed the High-PL or Low-PL MD. Microalgae (*Nannochloropsis gaditana* B3 strain and *Tisochrysis lutea*) were added to the tank water in the same amounts as indicated above. Temperature, salinity and oxygen were 20.0 ± 0.1°C, 33.5 ± 0.1 and 5.6 ± 0.2 ppm, respectively. Larvae were sampled at 3, 6, and 8 dph (8:00) for measuring larval somatic growth, biochemical composition and for gene expression analysis. Larval DM was determined as described in Fernández-Díaz et al. (2001). For lipid determination, three pools of larvae from each tank (300 larvae in each pool) were randomly collected, washed with clean seawater and ammonium formiate solution (1% w/w), frozen in liquid nitrogen and kept at −80°C until analysis. For RNA isolation, one pool of larvae per tank (50 larvae in each larval pool) were randomly collected at 3, 6 and 8 dph using a 350 μm mesh net, washed with DEPC water, euthanized with an overdose of tricaine methane sulphonate (MS-222), snap-frozen in liquid nitrogen and stored at −80°C until RNA isolation. For histological analysis, 30 larvae from each tank were randomly collected at 8 dph, euthanized with an MS-222 overdose and fixed in 10% v/v buffered formaldehyde at 4°C overnight and preserved in ethanol 70% until histological processing. Four samples (25 mg DM) of diets that were collected throughout the experimental period were also analyzed.

All procedures were authorized by the Bioethics and Animal Welfare Committee of IFAPA and given the registration number 06-11-15-337 by the National authorities for regulation of animal care and experimentation.

### Lipid and Histological Analysis

Lipid analysis and histological methods have been previously described (Hachero-Cruzado et al., 2014; Roman-Padilla et al., 2017). Briefly, TL was extracted using the chloroform:methanol method and gravimetrically quantified. LC were separated by one dimensional double development high performance thin layer chromatography (HPTLC), visualized by charring after dipping in cupric acetate in 3% phosphoric acid, and quantified by densitometry. FA methyl esters (FAME) were determined by acid-catalyzed transmethylation of TL extracts and quantified by gas chromatography. For histology, larvae were dehydrated through an ethanol series, embedded in paraffin wax, sectioned using a rotatory microtome and stained with Harris hematoxylin and eosin (H-E). A semi-automatic quantitative analysis was used to determine the percentage of fat deposits in the intestinal epithelium and hepatocytes (Roman-Padilla et al., 2017). Results are shown as the percentage of the total area in the tissue covered with lipid droplets.

### RNA Isolation and Gene Expression Analysis

Homogenization of the larval pools (*n* = 3 pools; 14–16 larvae/pool ∼40–50 mg) was carried out in the Fast-prep FG120 instrument (Bio101) using Lysing Matrix D (Q-Bio- Gene) for 40 s at speed setting 6. Total RNA isolation, RNA quantification and quality control were carried as previously described (Hachero-Cruzado et al., 2014). Expression analyses were performed in an OpenArray^®^ Real-Time PCR platform (Life Technologies, Carlsbad, United States) using plates with a format of 112 (assays) × 24 (samples). Overall, 108 assays targeted transcripts related to lipid metabolism: 28 transcripts (6 duplicates) involved in lipid transport, 13 (1 duplicate) involved in lipid signaling, 41 (8 duplicates) involved in glycerolipid metabolism and 26 (3 duplicates) involved in glycerophospholipid metabolism. Moreover, 1 transcript not involved lipid pathways (*cstf1*) and 3 putative reference genes for normalization (*ub52*, *eef1a1*, *gapdh2*) (Infante et al., 2008) were included (Supplementary Table S1). Primers and probes were designed using the custom Taqman Assay Design Tool (Life Technologies) following the standard parameters recommended by the manufacturer (Supplementary Table S1). For the analysis, total RNA (2 μg) from each sample was reverse-transcribed using the High Capacity cDNA Reverse Transcription Kit (Thermo Fisher) in accordance with the manufacturer’s protocol. The OpenArray^®^ Real-Time PCR Instrument (Life technologies) was used to run the Taqman assays using the TaqMan^®^ OpenArray^®^ Real-Time PCR Master Mix (Thermofisher). The thermal cycling consisted of a 10 min activation period at 95°C followed by 40 cycles of a two steps thermal profile of 15 s at 95°C denaturation and 60 s at 60°C for combined annealing-extension. For gene expression analysis, raw data were imported in Datassistv3.01 software and Ct values exported and analyzed according to the comparative Ct method using the geometric mean of *ub52 and gapdh2* reference genes for normalization (Vandesompele et al., 2002).

### Statistical Analysis

Data are presented as mean ± SD. All statistical analyses were considered significant when *P* < 0.05. All data were checked for normal distribution (Kolmogorov–Smirnov test) and homogeneity of variances (Levene’s test). When a normal distribution and/or homogeneity of the variances were not achieved, data were compared using a Mann–Whitney test. Log transformation was applied to analyze gene expression data. Arcsine transformations were applied to analyze FA percentages and histological analysis of surface covered by lipid vacuoles.

To assess statistical differences in the dietary lipid components, SGR, metamorphic index and in the percentage of lipid accumulation in histological sections a Student‘s *t*-test was used. The effect of diet on DM, gene expression, lipid class and FA composition was analyzed using a two-way ANOVA with factors “diet” and “age.” *Post hoc* Tukey test was performed when significant differences were found for “age.” Principal Component Analysis (PCA) was used to show the relationship between the dietary treatments (Low-PL and High-PL) and development (3, 6 and 8 dph) for gene expression, lipid class or FA composition.

ANOVA and *t*-tests were performed using SPSS statistics version 22 software (IBM, Armonk, United States) and PCAs and heatmap analyses were carried out using Clustvis (Metsalu and Vilo, 2015).

## Results

### Biochemical Characterization of Diets

Analysis of the proximal composition of MD indicated that High-PL had higher total PL and PC and lower TAG than the Low-PL diet. Small differences in FA profiles between MD were found. A higher content of palmitic acid (PA, 16:0) and arachidonic acid (ARA, 20:4n-6) and reduced levels of stearic acid (SA, 18:0), 16:1n-9, cetoleic acid (CA, 22:1n-11), eicosapentanoic acid (EPA, 20:5n-3), docosapentaenoic acid (DPA, 22:5n-3), and docosahexaenoic acid (DHA, 22:6n-3) was found in the High-PL compared to the Low-PL MD (Table 2).

### Long-Term Effects of MD Supply on Fish Performance and Survival

Larval growth as determined by DM is depicted in Figure 1. Larvae fed the High-PL had a statistically significant higher DM than larvae fed Low-PL at 8 dph (Student’s *t* test; *P* < 0.05). Larvae from the LP group had a higher DM from 3 to 29 dph than those fed with MD (two-way ANOVA, *P* < 0.05). For a more detailed analysis of those differences in growth, daily growth rates were compared by different developmental periods: 2–8 dph during MD supply, 8–3 dph after *Artemia* supply before metamorphosis, 13–20 during metamorphosis and 20–29 dph after metamorphosis completion (Table 3). Interestingly, LP only had higher SGR values than MD from 3 to 8 dph. In this period SGRs were also higher for larvae fed with High-PL compared to Low-PL (2.16 vs. −0.98% d^–1^). After *Artemia* were supplied to all experimental groups in pre-metamorphosis (8–13 dph) and metamorphosis (from 13 to 20 dph), a compensatory growth was observed in larvae fed MD with statistically significant higher SGR in MD treatments than LP group (Table 3). Metamorphic progress was clearly affected by diet and larvae from the LP group commenced and completed metamorphosis earlier that those fed with MD (Figure 1). No differences in metamorphosis progress were found between MD. Estimated survival did not differ between groups and was 58 ± 4, 58 ± 10, and 41 ± 10% for LP, High-PL and Low-PL, respectively, at the end of the trial.

**TABLE 3:** Specific growth rates (SGR) for each dietary treatment during four different periods (2–8: MD supply; 8–13: artemia feeding before metamorphosis; 13–20: metamorphosis; and 20–29 dph: post-metamorphosis).

	Dietary treatments			Statistics	
	LP	Low-PL	High-PL	MD vs. LP	High-PL vs. Low-PL
3–8 dph	18.19 ± 0.37	−0.98 ± 1.91	2.16 ± 1.23	***	*
8–13 dph	23.03 ± 0.78	33.45 ± 5.61	29.22 ± 5.52	*	ns
13–20 dph	15.92 ± 1.48	20.24 ± 4.05	22.07 ± 2.60	*	ns
20–29 dph	10.85 ± 0.75	12.01 ± 2.38	10.20 ± 2.39	ns	ns

Values are expressed as mean ± SD (n = 3). Significance for Student‘s t-tests between MD vs. LP and High-PL vs. Low-PL diets for each period are shown (ns, not-significance; *P < 0.05; **P < 0.01; ***P < 0.001).

### Short-Term Effects of MD Supply on Growth, Biochemical Characterization and Expression Profiles

#### Larval Performance and Lipid Composition

**FIGURE 2:**
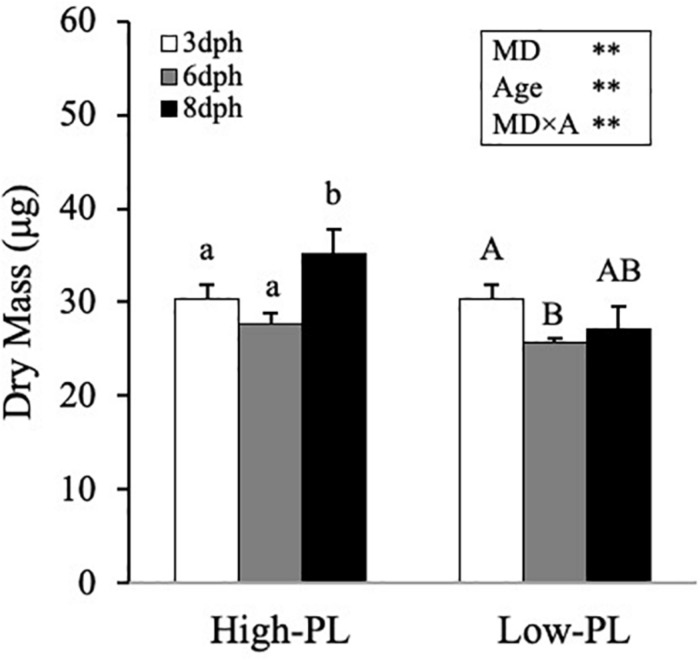
Growth (DM) of Senegalese sole larvae fed MD with high PL (High-PL) or low PL content (Low-PL). Data are presented as mean ± SD (*n* = 3). Results of two-way ANOVA are presented in the square. Different letter above bars indicates significant differences between sampling points (One-way ANOVA; *P* < 0.05). *P < 0.05; **P < 0.01; ***P < 0.001.

Once MD were confirmed as suitable feed in larvae with no differences in survival rates, a second trial was carried to compare the responses triggered in larvae fed these MD with different levels in PL. In common with the first trial, in the second trial (from 3 to 8 dph), larvae fed with High-PL had a higher DM (Figure 2) and grew faster than Low-PL (*P* < 0.005). SGR values were 2.89 ± 1.24 and −1.33 ± 0.51 for High-PL and Low-PL, respectively. It should be noted that before external feeding (3 dph), yolk sac was almost exhausted (Supplementary Figure S1), confirming that internal reserves for lipids could not interfere in our biochemical analysis at 6 and 8 dph and MD should be considered as the main source of lipids.

Lipid profiles of larvae fed MD during development are depicted in Table 4 and Supplementary Figure S2. FA multivariate analyses separated lecitotrophic larvae (3 dph) from exotrophic larvae (6 and 8 dph), and a small separation was found between larvae fed with High-PL and Low-PL (Supplementary Figures S2C,D). However, PCA and heatmap analyses using lipid classes did not separate samples by age (3, 6, 8 dph) or diet (lecitothrophic, High-LP, Low-LP) (Supplementary Figures S2A,B).

**TABLE 4:** Lipid classes (in % DM), and FA composition (in μg FA mg DM^–1^) in *Solea senegalensis* lecitotrophic larvae (3 dph) and 6 and 8 dph larvae fed MD with high PL (High-PL) or low (Low-PL) PL content.

	Age				Statistics		
	3 dph	MD	6 dph	8 dph	age	MD	age × MD
PC	8.1 ± 0.2	High-PL	5.8 ± 0.4	6.6 ± 0.9	3 > 6,8		
		Low-PL	5.2 ± 0.3	6.3 ± 1.1			
PE	3.8 ± 0.7	High-PL	3.5 ± 0.2	4.4 ± 0.8			
		Low-PL	3.3 ± 0.5	4.2 ± 1.0			
PI	1.2 ± 0.2	High-PL	1.0 ± 0.2	1.1 ± 0.1			
		Low-PL	1.0 ± 0.2	1.3 ± 0.2			
PS	1.0 ± 0.3	High-PL	1.0 ± 0.3	1.1 ± 0.1			
		Low-PL	1.3 ± 0.7	1.0 ± 0.3			
CHO	1.6 ± 0.2	High-PL	1.4 ± 0.1	1.5 ± 0.0		*	
		Low-PL	1.5 ± 0.1	1.9 ± 0.3			
TAG	2.9 ± 0.4	High-PL	0.6 ± 0.2	0.5 ± 0.0	3 > 6,8	*	*
		Low-PL	0.7 ± 0.3	1.9 ± 0.7			
MA (14:0)	1.6 ± 0.1	High-PL	1.5 ± 0.1	1.4 ± 0.2		*	
		Low-PL	1.9 ± 0.2	1.7 ± 0.2			
PA (16:0)	14.1 ± 3.0	High-PL	10.1 ± 0.6	10.3 ± 1.1			
		Low-PL	12.5 ± 2.4	9.8 ± 1.9			
SA (18:0)	6.0 ± 0.9	High-PL	5.7 ± 0.3	6.1 ± 0.5			
		Low-PL	7.2 ± 1.0	6.0 ± 0.5			
Total saturated	22.3 ± 4.1	High-PL	17.9 ± 0.9	18.4 ± 2.0			
		Low-PL	22.3 ± 3.7	18.1 ± 2.5			
(16:1n-9)	3.8 ± 0.7	High-PL	1.7 ± 0.0	1.6 ± 0.4	3 > 6,8		
		Low-PL	2.2 ± 0.5	1.7 ± 0.4			
OA (18:1n-9)	6.0 ± 1.7	High-PL	5.6 ± 0.4	5.6 ± 0.5			
		Low-PL	6.7 ± 1.8	5.3 ± 1.9			
GA (20:1n-9)	0.9 ± 0.2	High-PL	1.0 ± 0.1	1.1 ± 0.1			
		Low-PL	1.2 ± 0.2	1.3 ± 0.3			
CA (22:1n-11)	0.26 ± 0.01	High-PL	0.6 ± 0.0	0.6 ± 0.1		*	
		Low-PL	1.0 ± 0.2	1.0 ± 0.3			
Total monounsaturated	10.8 ± 2.5	High-PL	8.3 ± 0.5	8.3 ± 1.0			
		Low-PL	10.1 ± 2.5	8.3 ± 2.6			
LA (18:2n-6)	1.2 ± 0.2	High-PL	2.4 ± 0.1	3.0 ± 0.2	3 < 6,8	*	
		Low-PL	2.3 ± 0.6	2.9 ± 0.9			
EA (20:2n-6)	0.7 ± 0.1	High-PL	0.5 ± 0.0	0.5 ± 0.0		*	
		Low-PL	0.6 ± 0.1	0.6 ± 0.1			
ARA (20:4n-6)	2.23 ± 0.16	High-PL	2.0 ± 0.0	2.3 ± 0.2			
		Low-PL	2.6 ± 0.4	2.4 ± 0.3			
Total (n-6) PUFA	5.05 ± 0.29	High-PL	5.8 ± 0.1	6.6 ± 0.4			
		Low-PL	6.4 ± 1.2	6.8 ± 0.9			
ALA (18:3n-3)	0.36 ± 0.03	High-PL	0.4 ± 0.0	0.4 ± 0.1			
		Low-PL	0.5 ± 0.1	0.5 ± 0.2			
EPA (20:5 n-3)	3.20 ± 0.67	High-PL	2.2 ± 0.1	2.2 ± 0.4			
		Low-PL	2.6 ± 0.5	2.0 ± 0.4			
DHA (22:6 n-3)	19.29 ± 4.07	High-PL	14.5 ± 0.9	14.1 ± 2.2			
		Low-PL	17.3 ± 3.6	12.5 ± 1.8			
Total (n-3) PUFA	22.86 ± 4.76	High-PL	17.2 ± 0.8	16.7 ± 2.7			
		Low-PL	20.4 ± 4.1	15.0 ± 2.4			
Total PUFA	27.91 ± 5.05	High-PL	23.0 ± 1.0	23.4 ± 2.9			
		Low-PL	26.8 ± 5.1	21.8 ± 3.1			

Statistic results for two-way ANOVA (age, MD and interaction age × MD) are shown when significant (*P < 0.05). Post hoc Tukey results for age are shown. Values are expressed as mean (n = 3). Results of Two-way ANOVA analysis for the factors dietary treatments (diet) and age (3, 6 and 8 dph; t) and their interactions (age × diet) are also shown. PC, phosphocholine; PE, phosphatidylethanolamine; PI, phosphatidylinositol; PS, phosphatidylserine; TPL, total polar lipids; CHO, cholesterol; TAG, triacylglycerol; MA, Myristic acid; PA, palmitic acid; SA, stearic acid; OA, oleic acid; GA, gadoleic acid; CA, cetoleic acid; LA, linoleic acid; EA, eicosadienoic acid; ARA, arachidonic acid; ALA, α-linolenic acid; ETA, eicosatetraenoic acid; EPA, eicosapentaenoic acid; DPA, docosapentaenoic acid; DHA, docosahexaenoic acid.

Two-way ANOVA analysis showed higher contents of PC, TAG and monounsaturated FA 16:1n-9 for lecitotrophic larvae, and a higher content of linoleic acid (LA, 18:2n-6) for the exotrophic larvae. Comparisons of MD revealed that larvae fed with High-PL had reduced TAG and cholesterol (CHO) levels compared to the Low-PL group. Regarding the FA profile, myristic (MA, 14:0), cetoleic acid (CA, 22:1n-11) and eicosadienoic acid (EA, 20:2 n-6) were reduced and linolenic acid (LA,18:2n-6) was increased in the High-PL group compared to the Low-PL group (Table 4).

#### Histological Observations

**FIGURE 3:**
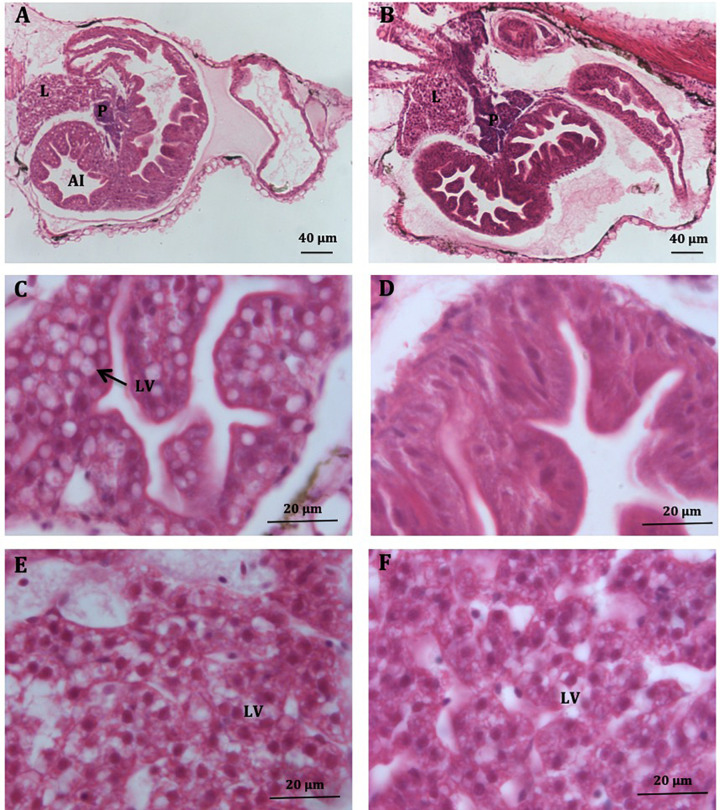
Histological sections of *S. Senegalensis* larvae at 8 dph. General view of the digestive system in larvae fed Low-PL **(A)** and High-PL **(B)** diets. **(C,D)** Detail of anterior intestine of larvae fed Low-PL **(C)** and High-PL **(D)** diets. Note the increase of lipid vacuoles in panel **(C)**. **(E,F)** Livers of Low-PL **(E)** and High-PL **(F)**. L, liver; P, exocrine pancreas; AI, anterior intestine; LV, lipids vacuoles. Scale is indicated.

Histological observations indicated that the occupation by lipid vacuoles of the anterior intestine surface was higher in Low-PL larvae (18.9%) compared to High-PL larvae (3.1%) (Figure 3). The percentage of larvae with intestinal lipid vacuoles was also higher in the Low-PL (55%) than High-PL (11%) group. The liver in larvae fed with both MD had a typical appearance with hepatocytes arranged in cords with a prominent central nucleus and an acidophilic cytoplasm containing lipid vacuoles (neutral lipids) (Figures 3E,F). No significant differences in lipid vacuoles were detected between MD (9.4% for Low-PL and 8.7% for High-PL).

#### Gene Expression Analysis

**FIGURE 4:**
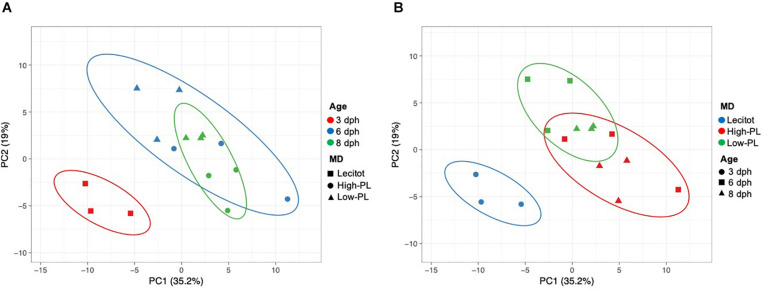
Principal Component Analysis (PCA) plot based on expression levels of 97 assays analyzed using OpenArray^®^ platform for sampling points (3, 6, and 8 dph) and nutritional status (lecitotrophic stage (lecitot) and MD (high-PL or low-PL). Points are surrounded by ellipses based on factors age **(A)** or MD **(B)**. Prediction ellipses indicate 85% confidence.

**FIGURE 5:**
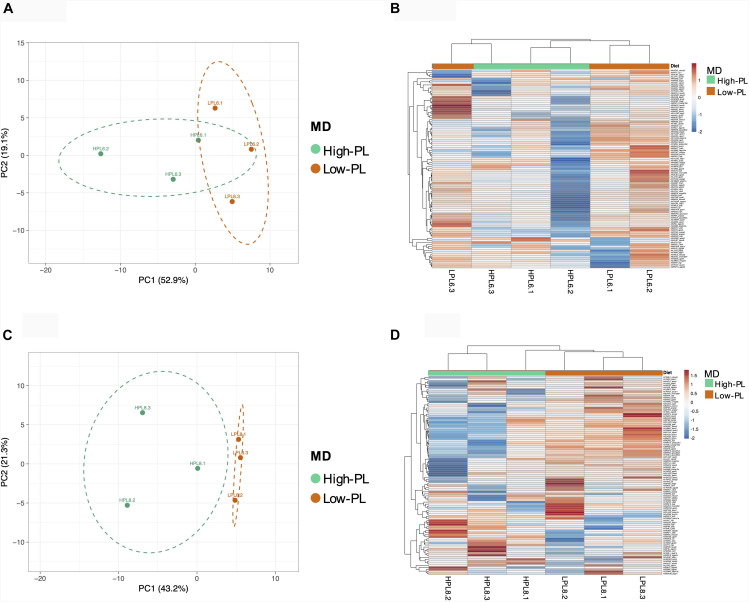
Principal Component Analysis (PCA) and heatmaps based on expression levels of 97 assays analyzed using OpenArray^®^ platform; for samples of 6 **(A,B)** and 8 dph **(C,D)**. Points are surrounded by ellipses based on MD (high-PL or low-PL). Prediction ellipses indicate 85% confidence.

Multivariate PCA analysis using the transcript levels of 97 assays that successfully amplified in the OpenArray analysis clearly separated the lecitotrophic larvae (3 dph) from the exotrophic larvae (6 and 8 dph) (Figure 4). In contrast, no clear separation of transcript levels was observed between High-PL and Low-PL (Figure 4B). When multivariate analyses were carried out by age (6 or 8 dph), larvae fed High-PL and Low-PL larvae were clearly separated only at 8 dph (Figure 5).

**FIGURE 6:**
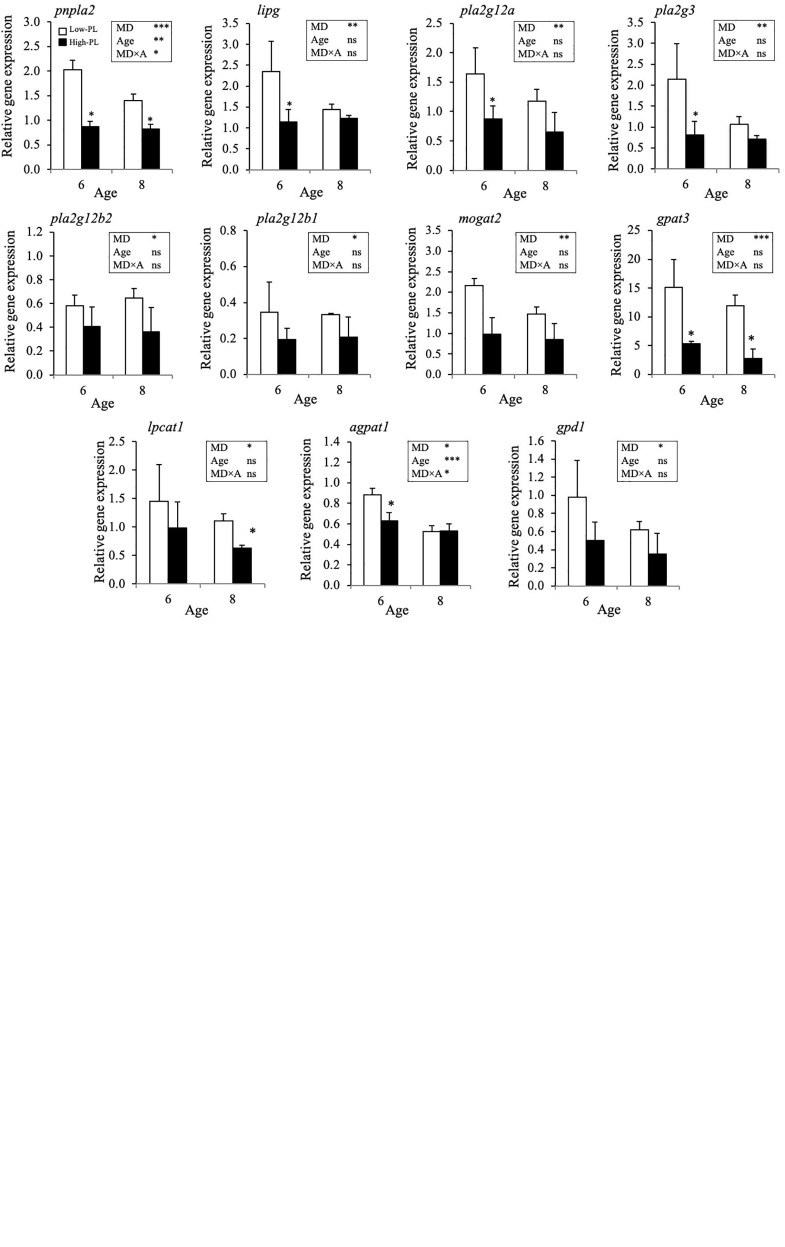
Relative gene expression level of transcripts significantly regulated by MD (Low-PL, white bars; High-PL, black bars) in pelagic larvae at 6 and 8 dph (Two-way ANOVA; *P* < 0.05). Results of two-way ANOVA are presented in the square. Data were expressed as the mean fold change (mean ± SD, *n* = 3) from the calibrator group (3 dph). Asterisk above bars denotes significant differences among MD for 6 or 8 dph larvae (One-way ANOVA; *P* < 0.05). ns, not significant; ^∗^*P* < 0.05; ^∗∗^*P* < 0.01; ^∗∗∗^*P* < 0.001.

A detailed analysis of expression data (two-way ANOVA) identified 15 differentially expressed transcripts (DET) during development, three up-regulated (*gpat3*, *lpin1*, *pnpl2*) and twelve down-regulated (*apob2*, *apoc1*, *apoc2*, *apod5*, *lrp8*, *agpat1*, *agpat3*, *pcyt1a*, *pcyt2*, *cept1*, *ptdss1a*, *cds2*) transcripts (Supplementary Table S2). Moreover, when the effect of diets was tested at 6 and 8 dph, 23 DETs were identified (Figure 6). Apolipoproteins (*apoa4Aa1*, *apoa4Ba3, apoa4Ba4, apob1, apoc1, apoc2, apoe1*, *and, apoe2*), lipases (*pnpla2*, *lipg*), phospholipases (*pla2g12a*, *pla2g12b1*, *pla2g12b2*, *pla2g3*), acyltransferases (*mogat2*, *gpat3, lpcat1, agpat1*), glycerol-3-phosphate dehydrogenase 1 (*gpd1*), CDP-diacylglycerol synthase 2 (*cds2*) and the transmembrane protein 269 (*tmem269*) with phosphatidyl serine synthase activity were up-regulated and only *apod5* and *apob2* were down-regulated in Low-PL compared to High-PL. The effect of MD was more notable at 8 dph.

## Discussion

Marine larvae require high levels of dietary PL for an optimal growth and survival (Cahu et al., 2009; Daprà et al., 2011; Azarm et al., 2013; Saleh et al., 2013; Zhao et al., 2013; De Santis et al., 2015; Taylor et al., 2015; Cai et al., 2017; Feng et al., 2017). Studies of PL requirements in fish larvae have been limited by the fact that live prey (LP) do not accumulate these structural compounds making it difficult to establish their nutritional requirements. The use of MD represents an attractive approach to overcome this limitation since their formulation can be easily modified and pelagic sole larvae accept these inert diets from mouth opening. Currently, main protocols adopted for sole larval rearing are based on MD and live prey co-feeding from mouth-opening until weaning. In contrast, MD supplied alone for a long term have low survival rates (Engrola et al., 2007, 2009; Pinto et al., 2018). In this study, we investigated how different dietary PL levels affect pelagic larval stages of sole, a short period of high energy demand due to the imminent metamorphosis (Yufera et al., 1999; Cañavate et al., 2006). The main effects of PL levels were evaluated at 6 and 8 dph to avoid interference from yolk sac reserves, which were observed to be almost fully depleted by 3 dph as previously reported (Roman-Padilla et al., 2016a, b, 2018). As expected, larvae supplied with live prey grew faster than those fed with MD. However, survival remained unaffected indicating that MD could be useful to investigate the effect of micronutrients when supplied for short-periods in pelagic sole larvae as previously suggested (Gamboa-Delgado et al., 2011; Jimenez-Fernandez et al., 2015). Sole larvae require an adaptation period to inert diets that is inversely proportional to size and hence co-feeding protocols appear as the most adequate strategy in long-term procedures and for the industry (Engrola et al., 2007; Pinto et al., 2018). However, the use of MD for short periods provides a suitable experimental window to investigate nutritional requirements and larval physiology in this species.

Larvae fed with High-PL grew faster than low-PL MD in our two independent trials demonstrating the importance of PL for larval growth. Interestingly, the replacement of MD by *Artemia* after 8 dph triggered compensatory growth and by the end of the trial there were no differences in survival or mass compared to a reference diet based on LP indicating physiological compensation of the larvae and the full capacity of development when optimal conditions were restored. The shift to an optimal diet enhanced energy accumulation so that the Senegalese sole underwent successful metamorphosis and recovered mass and were no different from the LP diet at the end of the trial. Jimenez-Fernandez et al. (2015) observed similar effects on growth and development during the period that we supplied MD in our trial. Overall, our data demonstrate that pelagic sole larvae can deal with suboptimal levels of dietary PL by reducing transiently growth but recovering full performance if a shift to an optimal composition occurs. This further supports the suitability of our approach to evaluate the physiological effect of dietary treatments.

One major effect of PL dietary treatments was the presence of vacuoles in the intestine. According to our previous study, using oil red staining of cryostat sections, larvae tend to accumulate neutral lipids in the anterior intestine within large vacuoles (Hachero-Cruzado et al., 2014). Others authors using haematoxylin and eosin staining have also reported intestinal vacuolization linked to neutral lipid accumulation (Ribeiro et al., 1999; Gisbert et al., 2005; Morais et al., 2006; Daprà et al., 2011). Lipid vacuoles were far more abundant in the intestine of larvae fed with Low-PL than High-PL in our study (18.9% in larvae fed LPL vs. 3.05% for HPL larvae) and this observation coincides with the higher TAG, CHO and cetoleic acid (CA, 22:1n-11) detected in LPL larvae, suggesting accumulation of dietary neutral lipids in the intestine. These findings may be explained by ineffective lipid mobilization and transport from the intestine to internal tissues when dietary PL/TAG ratio is low. The results reinforce the importance of balanced dietary PL for efficient lipid transport (Fontagné et al., 1998; Olsen et al., 1999; Salhi et al., 1999; Gisbert et al., 2005; Cahu et al., 2009; Carmona-Antoñanzas et al., 2015).

Gene expression data indicated a major effect of development from day 3 to 8 dph that is in alignment with the maturation of the digestive system and metabolism (Manchado et al., 2008; Gamboa-Delgado et al., 2011; Hachero-Cruzado et al., 2014). However, the effects of the dietary treatments were still observable at 8 dph with a wide set of apolipoproteins-encoding genes differentially regulated. A previous study in early larval stages of sole indicated that animals responded to dietary TAG levels under adequate levels of PL by activating the transcription of several apolipoproteins to facilitate lipid mobilization in the intestine (Hachero-Cruzado et al., 2014; Roman-Padilla et al., 2017). In this study, larvae fed with Low-PL had higher expression of the 8 genes encoding for main canonical apolipoproteins (types *apoA-IV*, apoB, apoC and apoE) than the High-PL group. This seems contradictory in the context of the accumulation of dietary lipids in vacuoles in the intestine of the Low-PL larvae. However, De Santis et al. (2015) reported that salmon fry fed a low PL diets also had up-regulated *apoa4* transcription without modifying intestinal lipid transport. These authors hypothesized that in salmon fry dietary lipids were absorbed into the enterocytes but not exported to the blood stream and this was associated with transcription of genes involved in chylomicron formation. Our results suggest a similar response to Low-PL in the intestine of the sole larvae, with a coordinated transcriptional activation of apolipoproteins induced by dietary lipids unable to promote an effective intestinal lipid transport probably due to the limited amounts of PL and failed lipoprotein assembly. In this sense, it is noteworthy the down-regulation of apolipoprotein *apob2* in larvae fed with Low-PL. This inverse pattern of regulation in relation to other apolipoproteins was not detected in our previous study (Hachero-Cruzado et al., 2014). Moreover, Daprà et al. (2011) found reduced *apob* expression in rainbow trout fry fed a PL-free diet compared to those fed PL-supplemented diets. However, they did not detect differences in expression for apolipoproteins *apoa1* and *apoa4*. Thus, the PL levels seem to positively regulate *apob2* expression in the intestine of sole larvae and this may be linked to chylomicron biosynthesis particularly since ApoB is an important structural component of chylomicrons (Iqbal and Hussain, 2009).

Together with the significance of PL regulating the synthesis and secretion of lipoproteins, it is assumed that fish larvae have a limited PL biosynthesis capacity and a major dependence on dietary supply (Tocher et al., 2008; Olsen et al., 2014; Carmona-Antoñanzas et al., 2015; De Santis et al., 2015; Feng et al., 2017). Results of the present study indicate that PL biosynthesis pathways were not fully developed at this early life stages. Sole larvae did not increase the transcription of key genes involved in *de novo* PL biosynthesis (ethanolamine phosphotransferase, *ept*; choline/ethanolamine phosphotransferase; *cpt*; phosphatidylethanolamine methyltransferase, *pemt*; or CDP-diacylglycerol inositol 3-phosphatidyltransferase, *cdipt*), despite differences in dietary PL ingested (0.73 vs. 4.97% DM). Only those transcripts involved in phosphatidylserine (PS) synthesis (CDP-diacylglycerol synthase (*cds2*) and transmembrane protein 269 (*tmem269*) responded to low dietary PL level. PS is a quantitatively minor PL, and constitute approximately 2–10% of total PL, differing between tissues (Vance and Steenbergen, 2005). About PS, it is interesting to note the clear dependence of gene expression of both transcripts involved in PS synthesis (*cds2* and *ptdss1a*) on sole larvae development (see Supplementary Table S2). So, we cannot discard that larval development was related with differences detected between dietary treatments. About the most pathways involved in PL synthesis, our results match with those reported for other fish species (Daprà et al., 2011; De Santis et al., 2015; Feng et al., 2017), which neither showed substantial changes in larval PL biosynthesis pathway with dietary PL level. However, we found changes in expression levels of other genes indirectly related with the synthesis of intermediate products of PL biosynthesis pathways (phosphatidic acid and DAG; *gpat3*, *agpat1*, *pnpla2*, *lipg* and *mogat2*) or with PL remodeling pathway (*pla2g12a, pla2g12b1*, *pla2g12b2*, *pla2g3*, and *lpcat1*). Phosphatidic acid is the precursor for CDP-DAG that is used in the synthesis of phosphatidylinositol (PI) and PS, and is dephosphorylated to diacylglycerol (DAG) for the synthesis of PC and phosphatidylethanolamine (PE) as well as TAG. Regarding PL remodeling, this pathway allows altered FA composition of individual PL by FA hydrolysis and re-esterification reactions. These combinations and permutations of FA attached to the *sn*-1 and *sn*-2 positions result in thousands of different PL species (Ridgway, 2016). These findings indicate that early sole larvae modulate the synthesis of PL intermediates and activate PL remodeling pathways when dietary PL levels are suboptimal but they fail in the regulation of key genes involved in *de novo* PL biosynthesis.

In summary, the present study demonstrates that dietary PL levels play a key role in growth of pelagic larvae in sole and they can deal with different dietary PL levels triggering an adaptive response to mobilize dietary lipids. While a low supply of PL delays growth, the larvae maintain viability and competence to trigger a compensatory growth when a shift in the diet occurs without affecting metamorphosis success or survival rates. The lipid accumulation in the intestine and the lack of efficient lipid mobilization mechanisms in spite of a coordinated activation of the gene expression related to PL intermediate synthesis, PL remodeling as well as several apolipoprotein transcripts are mainly responsible for the negative effects on growth. The lack of full functionality for PL biosynthesis pathways limits the lipid dietary uptake in these early sole larvae. These results are of relevance to optimize nutrition in sole larvae and provide a new marker (*apob2*) for monitoring specific responses to dietary PL.

## Data Availability Statement

The raw data supporting the conclusions of this article will be made available by the authors, without undue reservation.

## Ethics Statement

The animal study was reviewed and approved by Bioethics and Animal Welfare Committee of IFAPA. Written informed consent was obtained from the owners for the participation of their animals in this study.

## Author Contributions

IH-C and MM conceived and designed the experiments and wrote the manuscript. IT, AR-R, IH-C, and JR-P performed the experiments. IH-C, MM, and AR-R analyzed the data. All authors contributed to reagents, materials, and analysis tools. AR-R, IT, and JR-P contributed to additional writing and discussion.

## Conflict of Interest

The authors declare that the research was conducted in the absence of any commercial or financial relationships that could be construed as a potential conflict of interest.

## Supplementary Material

FIGURE S1 Yolk sac volumes (mm^3^) [mean ± standard deviation (SD)] in larvae reared during the first 4 days post-hatch (dph).

FIGURE S2 Principal Component Analysis (PCA) and heatmap plots based on lipid classes **(A,B)** and FA **(C,D)** for sole larvae fed High-PL and Low-PL at 6 and 8 dph, and lecithotrophic larvae (3 dph). Prediction ellipses indicate 85% confidence. PS, Phosphatidylserine; PE, phosphatidylethanolamine; CHO, cholesterol; PI, phosphatidylinositol; PC, phosphatidylcholine; TAG, triacylglycerol; PUFA, polyunsaturated fatty acids; MUFA, monounsaturated fatty acids; SAT, saturated fatty acids.

TABLE S1 Openarray plate design. Accession numbers of each transcript, gene name, gene description, Primers, Taqman probes and assay ID for the complete set of 112 assays are indicated. The transcripts are organized by categories (lipid Transport, lipid signaling, glycerolipid metabolism, glycerophospholipid metabolism and Gene expression and housekeeping). Accession numbers correspond to transcripts available in NCBI/EMBL/DDBJ, soleaDBv4.1 (ID starting by solea_v4.1) or SoleaDBv3 (ID starting by solea_v3.0). Those assays with the same Accession number should be considered as duplicates.

TABLE S2 Differentially expressed transcripts during development (3, 6 and 8 dph larvae) in OpenArray^®^ assay analysis (see Supplementary Table S1). Assay codes for genes analyzed, gene description, gene name and fold-change (mean ± SD, *n* = 3) are shown. Results of two-way ANOVA analysis (*post hoc* Tukey) for age factor are shown. Significant differences associated with the diet factor are shown in Figure 6.

## Abbreviations

agpat1: 1-acyl-sn-glycerol-3-phosphate acyltransferase alpha
apoa4: apolipoprotein A-IV
apob: apolipoprotein B
apod: apolipoprotein D
apoe: apolipoprotein C
apoe: apolipoprotein E
CDIPT: CDP-diacylglycerol inositol 3-phosphatidyltransferase
cds2: CDP-diacylglycerol synthase 2
CHO: cholesterol
CPT: CDP-choline-dependent
DET: differentially expressed transcripts
dph: days post-hatch
FA: fatty acids
gpat3: glycerol-3-phosphate acyltransferase 3
gpd1: glycerol-3-phosphate dehydrogenase 1
High-PL: high phospholipid levels
lipg: endothelial lipase
Low-PL: low phospholipid levels
LP: live preys
LPCATs: lysoPC acyltransferases
lysoPC: lysophosphatidylcholine
MD: microdiets
mogat2: monoacylglycerol O-acyltransferase 2
PC: phosphatidylcholine
PE: phosphatidylethanolamine
PEMT: phosphatidylethanolamine N-methytransferase
PI: phosphatidylinositol
PL: phospholipids
PLA2: phospholipase A2
pnpla2: patatin like phospholipase domain containing 2
PS: phosphatidylserine
TAG: triacylglycerol
tmem269: transmembrane protein 269.
