# Supplementary figures and images for: Assessment of Growth, Lipid Metabolism and Gene Expression Responses in Senegalese Sole Larvae Fed With Low Dietary Phospholipid Levels
Source: Front Physiol. 2020 Sep 30;11:572545. doi: 10.3389/fphys.2020.572545 (PMC7569605; doi:10.3389/fphys.2020.572545)

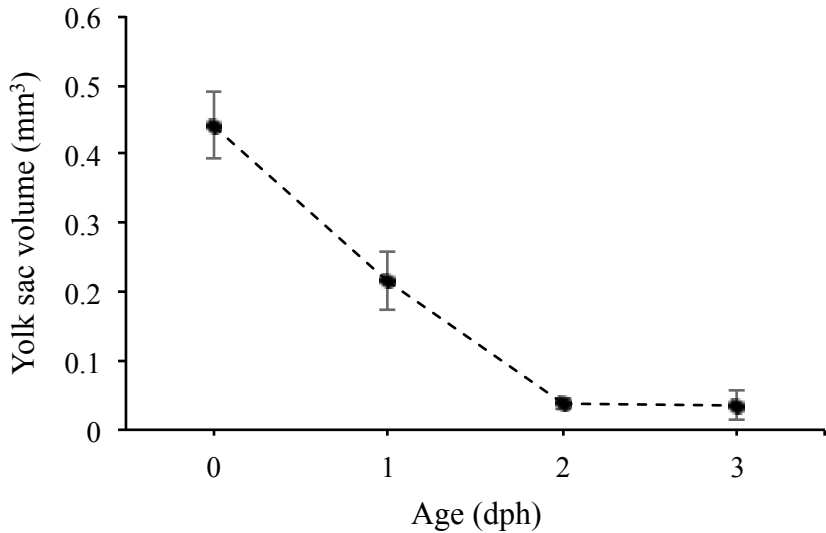

Supplement: FIGURE S1 — Yolk sac volumes (mm3) [mean ± standard deviation (SD)] in larvae reared during the first 4 days post-hatch (dph). [file Data_Sheet_1.PDF]

A

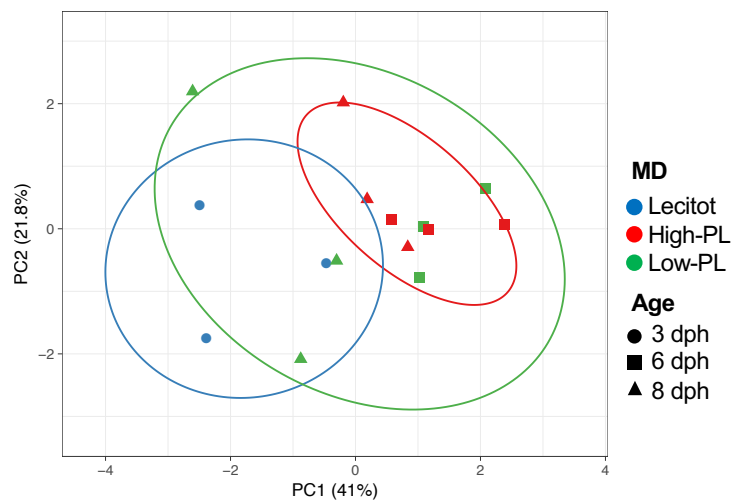

B

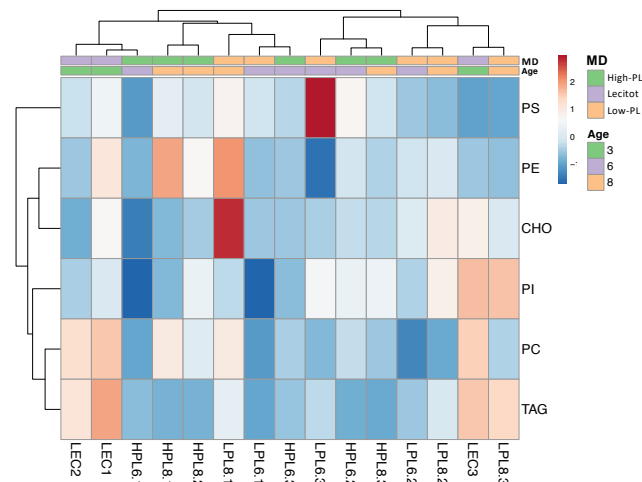

C

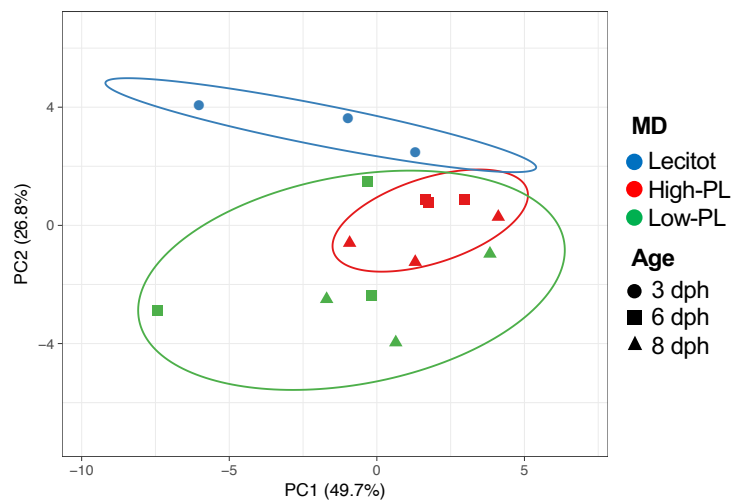

D

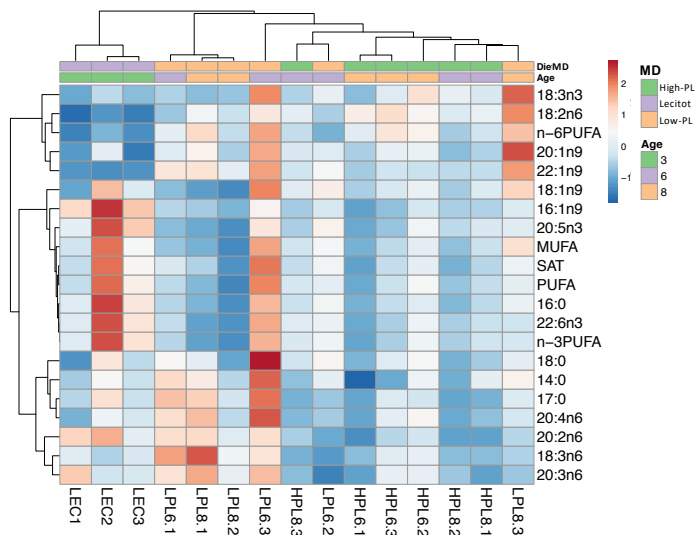

Supplement: FIGURE S2 — Principal Component Analysis (PCA) and heatmap plots based on lipid classes (A,B) and FA (C,D) for sole larvae fed High-PL and Low-PL at 6 and 8 dph, and lecithotrophic larvae (3 dph). Prediction ellipses indicate 85% confidence. PS, Phosphatidylserine; PE, phosphatidylethanolamine; CHO, cholesterol; PI, phosphatidylinositol; PC, phosphatidylcholine; TAG, triacylglycerol; PUFA, polyunsaturated fatty acids; MUFA, monounsaturated fatty acids; SAT, saturated fatty acids. [file Data_Sheet_2.PDF]
