# Supplementary material for: Assessment of Growth, Lipid Metabolism and Gene Expression Responses in Senegalese Sole Larvae Fed With Low Dietary Phospholipid Levels
Source: Front Physiol. 2020 Sep 30;11:572545. doi: 10.3389/fphys.2020.572545 (PMC7569605; doi:10.3389/fphys.2020.572545)
Supplement: TABLE S2 — Differentially expressed transcripts during development (3, 6 and 8 dph larvae) in OpenArray® assay analysis (see Supplementary Table S1). Assay codes for genes analyzed, gene description, gene name and fold-change (mean ± SD, n = 3) are shown. Results of two-way ANOVA analysis (post hoc Tukey) for age factor are shown. Significant differences associated with the diet factor are shown in Figure 6. [file Table_2.DOCX]

Supplementary Table 2. Differentially expressed transcripts during development (3, 6 and 8 dph larvae) in OpenArray^®^ chip analysis (Hachero-Cruzado et al. 2014, Suppl. file 1). Assay codes for genes analyzed, gene description, gene name and fold-change (mean ± SD, n = 3) are shown. Results of two-way ANOVA analysis (post-hoc Tukey) for age factor are shown. Significant differences associated with the diet factor are shown in Figure 6.

| Assay ID | Gene name | Fold-change | | | |  |
| --- | --- | --- | --- | --- | --- | --- |
|  |  | 3 dph | MD | 6 dph | 8 dph |  |
| AIFASMX | *apob2* | 1.08±0.48 | High-PL | 0.31±0.15 | 0.53±0.13 | 3 > 8 > 6 dph |
|  |  |  | Low-PL | 0.12±0.04 | 0.30±0.14 |  |
| AIGJQS5 | *apoc1* | 1.08±0.47 | High-PL | 0.15±0.13 | 0.32±0.08 | 3 > 6 = 8 dph |
|  |  |  | Low-PL | 0.35±0.07 | 0.49±0.08 |  |
| AIHSOZD | *apoc2* | 1.03±0.30 | High-PL | 0.37±0.30 | 0.70±0.11 | 3 = 8 > 6 dph |
|  |  |  | Low-PL | 0.68±0.09 | 1.05±0.27 |  |
| AIKALBT | *apod5* | 1.02±0.22 | High-PL | 0.28±0.03 | 0.20±0.05 | 3 > 6 > 8 dph |
|  |  |  | Low-PL | 0.22±0.01 | 0.13±0.03 |  |
| AIX0079 | *lrp8* | 1.05±0.39 | High-PL | 0.39±0.13 | 0.34±0.14 | 3 > 6, 8 dph |
|  |  |  | Low-PL | 0.58±0.26 | 0.24±0.03 |  |
| AIS08JC | *agpat3* | 1.04±0.35 | High-PL | 0.40±0.13 | 0.32±0.10 | 3 > 6, 8 dph |
|  |  |  | Low-PL | 0.57±0.14 | 0.32±0.02 |  |
| AIX008A | *agpat1* | 1.02±0.22 | High-PL | 0.63±0.08 | 0.53±0.07 | 3 > 6 > 8 dph |
|  |  |  | Low-PL | 0.88±0.07 | 0.52±0.06 |  |
| AIX0078 | *gpat3* | 1.07±0.45 | High-PL | 5.32±0.47 | 2.78±1.66 | 6 = 8 > 3 dph |
|  |  |  | Low-PL | 15.10±4.89 | 11.91±1.84 |  |
| AIGJQS8 | *pcyt1a* | 1.00±0.11 | High-PL | 0.49±0.14 | 0.47±0.04 | 3 > 6 = 8 dph |
|  |  |  | Low-PL | 0.93±0.33 | 0.47±0.13 |  |
| AIHSOZG | *pcyt2* | 1.01±0.16 | High-PL | 0.44±0.12 | 0.36±0.04 | 3 > 6 = 8 dph |
|  |  |  | Low-PL | 0.53±0.17 | 0.33±0.04 |  |
| AIAAZX2 | *cept1* | 1.01±0.21 | High-PL | 0.63±0.18 | 0.36±0.09 | 3 > 6 > 8 dph |
|  |  |  | Low-PL | 0.77±0.05 | 0.47±0.09 |  |
| AICSWAK | *ptdss1a* | 1.00±0.11 | High-PL | 0.40±0.06 | 0.35±0.00 | 3 > 6 > 8 dph |
|  |  |  | Low-PL | 0.57±0.09 | 0.33±0.08 |  |
| AIN1FUJ | *cds2* | 1.01±0.16 | High-PL | 0.58±0.18 | 0.30±0.10 | 3 > 6 > 8 dph |
|  |  |  | Low-PL | 0.97±0.07 | 0.46±0.05 |  |
| AIT96PM | *lpin1* | 1.00±0.09 | High-PL | 2.08±1.43 | 1.17±0.16 | 3 = 8 < 6 dph |
|  |  |  | Low-PL | 2.58±0.93 | 1.11±0.17 |  |
| AI20TW4 | *pnpl2* | 1.01±0.20 | High-PL | 0.87±0.11 | 0.82±0.09 | 3 = 8 < 6 dph |
|  |  |  | Low-PL | 2.03±0.19 | 1.39±0.14 |  |
